# Hemodynamic Challenges of Lower Extremity Lymphovenous Anastomosis: A Critical Reappraisal

**DOI:** 10.3390/jcm15041594

**Published:** 2026-02-18

**Authors:** Daihun Kang

**Affiliations:** 1 Department of Plastic and Reconstructive Surgery, Ewha Womans University Seoul Hospital, Seoul 07804, Republic of Korea; gpk1234567@ewha.ac.kr; 2 College of Medicine, Ewha Womans University, Seoul 07804, Republic of Korea

**Keywords:** lymphovenous anastomosis, lymphaticovenous anastomosis, lymphaticovenular anastomosis, lymphedema, lower extremity, hemodynamics, venous pressure, supermicrosurgery, microsurgery, Virchow triad

## Abstract

Lymphovenous anastomosis (LVA) has become an established microsurgical treatment for lymphedema, yet the hemodynamic basis for its efficacy in the lower extremity has not been rigorously examined. Most assessments of anastomotic function are performed in the supine position, where lymphatic pressure exceeds venous pressure, creating a favorable gradient for drainage. However, adults spend 16–18 h daily in upright postures, during which ankle-level venous pressure rises to 80–100 mmHg while lymphatic pressure increases only modestly. This pressure reversal raises questions about whether lower extremity LVA can function during routine daily activities. Several protective mechanisms have been proposed, including careful recipient venule selection, competent venous valves, and calf muscle pump assistance, yet these safeguards are inherently intermittent rather than continuous. Clinical data reveal progressive anastomotic patency decline over time, with some studies reporting 64% occlusion at two years, a pattern consistent with cumulative hemodynamic injury. Notably, clinical improvement sometimes persists despite declining patency, suggesting that concurrent conservative therapy or selection bias may contribute to observed outcomes. This review critically examines the postural hemodynamics relevant to lower extremity LVA, evaluates proposed protective mechanisms, and argues that the fundamental premise of sustained upright-posture drainage remains untested after three decades of clinical practice.

## 1. Introduction

Lymphovenous anastomosis (LVA) has been celebrated for more than three decades as a microsurgical solution for lymphedema, yet much of this enthusiasm has unfolded under hemodynamic conditions that rarely exist in everyday life [1]. While the literature has meticulously refined techniques, devices, and patency criteria, it has largely evaluated LVA in the supine position, where lymph-to-vein pressure gradients are inherently favorable and gravitational forces are minimized [2,3]. In contrast, during the upright posture that dominates daily activity, gravity reverses the pressure relationship between lymphatic vessels and veins—particularly in the lower extremity—creating conditions that should not only impair lymphatic drainage but even promote retrograde venous flow into lymphatic channels.

Despite these fundamental concerns, LVA has gained widespread acceptance as a physiological treatment for lymphedema, with high-volume centers reporting clinical success rates of 70–80% [1,4]. The procedure’s appeal lies in its elegant simplicity: microsurgical connections between obstructed lymphatic vessels and nearby venules bypass damaged lymph nodes and redirect lymph fluid directly into venous circulation [1,2]. This mechanism assumes that lymphatic pressure consistently exceeds venous pressure at the anastomosis site—an assumption validated primarily through intraoperative observations and postoperative assessments performed in supine patients [5]. What remains largely unexamined is whether this pressure relationship persists during the upright postures that constitute the majority of waking hours.

However, supine posture represents only 6–8 h of daily life. The remaining 16–18 h are spent upright—sitting, standing, and walking. During these activities, fundamental hemodynamic principles dictate dramatic reversal of the pressure relationship, particularly in the lower extremity where gravitational forces are maximal. The 120 cm hydrostatic column from ankle to heart generates venous pressures of 80–100 mmHg, while lymphatic pressure increases far less due to segmental flow and dense valvular compartmentalization, resulting in unfavorable gradients that should not only prevent lymphatic drainage but actively force venous blood retrograde into lymphatic channels [6,7,8,9].

This critical review focuses on lower extremity lymphedema, where hemodynamic challenges are most severe due to maximal gravitational forces. It challenges the mechanistic assumptions underlying LVA by examining pressure dynamics during upright posture, analyzing thrombogenic conditions at anastomosis sites, and evaluating alternative mechanisms for clinical improvement. The aim is to identify fundamental knowledge gaps that have persisted despite three decades of clinical experience and to propose research priorities essential for evidence-based patient counseling.

## 2. Hemodynamic Considerations

### 2.1. Postural Pressure Dynamics

**Figure 1:**
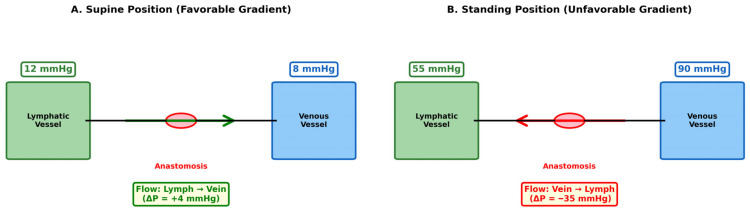
Pressure gradient dynamics at lymphovenous anastomosis in supine versus standing positions. (**A**) Supine position creates a favorable gradient: lymphatic pressure (12 mmHg) exceeds venous pressure (8 mmHg), permitting lymph-to-vein drainage (ΔP = + 4 mmHg). (**B**) Standing position reverses this relationship: venous hydrostatic pressure (90 mmHg) substantially exceeds lymphatic pressure (55 mmHg), creating an unfavorable gradient (ΔP = −35 mmHg) that theoretically opposes drainage and may promote venous reflux.

In the supine position, a modest but favorable pressure gradient of approximately 4 mmHg (lymphatic ~12 mmHg versus venule ~8 mmHg) permits lymph-to-vein drainage [10,11] (Figure 1A). However, this gradient reverses dramatically upon standing.

In contrast, upright posture introduces a hydrostatic component to dependent-limb venous pressure that increases with the vertical distance from the right atrium. Venous pressure at ankle level rises to 80–100 mmHg due to the approximately 120 cm hydrostatic column between the foot and heart [6,9,11]. The corresponding rise in intralymphatic pressure depends on the functional status of lymphatic valves, yet either scenario is unfavorable for LVA. If lymphatic valves are incompetent—as traditionally assumed in lymphedematous limbs—segmental compartmentalization is disrupted, and intralymphatic pressure may rise to an estimated 50–60 mmHg [12,13], still well below the venous pressure and leaving a 30–40 mmHg gradient that favors venous reflux into the lymphatic channels rather than lymphatic drainage into the veins (Figure 1B). However, Mackie et al. demonstrated that valve incompetence is rare in lymphedema, with retrograde lymph flow identified in only 3.7% of 566 patients and virtually absent in cancer-related lower limb lymphedema [14]. If lymphatic valves remain competent—as this evidence suggests—segmental compartmentalization is preserved, intralymphatic pressure during standing would be considerably lower than 50–60 mmHg, and the adverse pressure gradient would widen further. In short, incompetent valves make LVA unlikely to work; competent valves make it even less likely to work. The gradient depicted in Figure 1B therefore represents a conservative estimate, and the true hemodynamic disadvantage during upright posture may be substantially greater. Supine-position assessments, which constitute virtually all published evaluations of LVA function, fundamentally misrepresent the hemodynamic environment that these anastomoses must withstand during the 16–18 h of daily upright activity.

### 2.2. Proposed Protective Mechanisms and Their Limitations

Several anatomical and physiological mechanisms have been proposed to mitigate adverse postural hemodynamics in lower extremity LVA. A balanced appraisal requires acknowledging these protective mechanisms while recognizing their inherent limitations.

#### 2.2.1. Recipient Venule Selection

Recipient venule selection has been emphasized as a critical determinant of technical success. Visconti and colleagues proposed a flow-dynamic classification system, favoring venules with demonstrable outflow and minimal backflow [15]. Bianchi and colleagues developed a selection algorithm based on 1000 anastomoses, prioritizing outlet-pattern venules with competent valves [16]. Akita and colleagues recommended positioning anastomoses distal to functioning valves and avoiding venules with visible reflux on preoperative mapping [17]. Chen and colleagues advocated intraoperative evaluation of flow direction, selecting anastomosis sites where lymphatic pressure exceeds venous pressure [18].

Collectively, these strategies support the concept that careful recipient selection may reduce immediate backflow risk and improve early technical performance. However, most evidence derives from assessments conducted under supine conditions. Whether these selection criteria provide durable protection during chronic upright hemodynamic stress remains uncertain. Even when intact valves are present, valve function is not binary and may deteriorate under repeated hemodynamic challenges. The long-term competence of small venular valves at the anastomotic junction has not been systematically characterized.

#### 2.2.2. Muscle Pump Assistance

The calf muscle pump can reduce ambulatory venous pressure by propelling venous blood centrally during effective contractions [19]. In principle, such ambulatory pressure reductions may transiently improve the pressure gradient across LVA and facilitate drainage during active walking.

However, muscle pump effectiveness varies substantially across individuals and clinical contexts. Reduced mobility, pain, or heaviness are common in advanced lower extremity lymphedema, potentially diminishing pump efficacy precisely in patients who undergo LVA. Moreover, muscle pump benefits are realized only during active contraction cycles; prolonged standing, sitting, or sedentary periods—common in daily life—afford no such protection.

#### 2.2.3. Intermittent Protection Is Not Continuous Protection

While valve competence, muscle pump activity, and recipient optimization may reduce reflux risk during portions of the day, these mechanisms are by nature intermittent, raising the question of whether partial-day protection suffices for long-term anastomotic viability [2]. This concept can be framed as “time-in-unfavorable-gradient”: the cumulative duration during which local venous pressure likely exceeds lymphatic pressure such that antegrade lymph flow through the anastomosis is not mechanically favored. Whether anastomoses can tolerate substantial daily exposure to unfavorable gradients without progressive injury remains an open question.

## 3. Pathophysiological Considerations at the Anastomotic Site

Classical frameworks for thrombosis—such as Virchow’s triad—were developed to explain thrombus formation within blood vessels and are not directly applicable to lymphatic fluid itself, which contains substantially lower concentrations of coagulation factors than plasma [20,21]. However, the lymphovenous anastomosis creates a unique microenvironmental interface where blood and lymph meet, and where venous blood may reflux into the anastomotic junction during periods of unfavorable pressure gradients.

**Figure 2:**
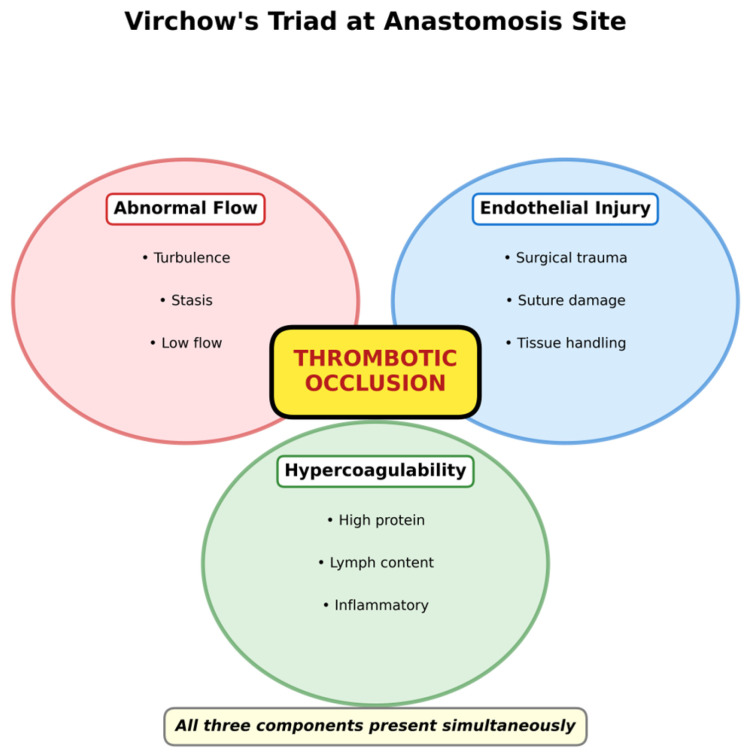
Application of Virchow’s triad to the lymphovenous anastomosis site. Two components—abnormal flow (stasis, turbulence during unfavorable pressure gradients) and endothelial injury (surgical trauma, suture placement)—are inherently present. The third component, hypercoagulability, becomes relevant only when venous blood refluxes into the anastomosis; lymph itself lacks platelets and coagulation factors. Thus, the thrombotic risk is primarily associated with periods of reversed pressure gradient permitting venous backflow.

The relevant application of Virchow’s triad pertains specifically to the venous/anastomotic side of LVA (Figure 2). First, stasis or low-flow states may develop in the recipient venule segment during periods when the pressure gradient is unfavorable; such low-shear conditions are well-recognized promoters of endothelial activation and thrombosis [21,22]. Second, endothelial perturbation is inherent to the procedure: surgical manipulation, suture placement, and the interface between lymphatic and venous endothelium introduce localized injury, while mismatch between lymphatic and venous wall composition may further contribute to abnormal healing responses [23]. Third, hypercoagulability becomes relevant only when venous blood—carrying platelets and coagulation factors—refluxes into the anastomosis; lymph itself lacks these components [23].

Rather than acute failure, lower extremity LVA may undergo gradual deterioration through cumulative, low-grade injury. Repeated episodes of unfavorable pressure gradients, transient stasis, and microtrauma at the anastomosis—occurring thousands of times during daily postural changes—could produce progressive endothelial dysfunction, subclinical microthrombosis, and reactive fibrosis. Chronic inflammation and mechanical stress at the junction are recognized stimuli for fibrotic remodeling in vascular tissues [24]. Over months to years, these incremental insults may narrow and ultimately occlude the anastomosis through gradual luminal encroachment, even in the absence of a single catastrophic thrombotic event.

This framework remains a testable hypothesis rather than established fact. Its value lies in motivating studies that quantify ambulatory venous pressures at recipient sites, characterize flow direction under various postures, and correlate hemodynamic exposure with long-term patency outcomes.

## 4. Clinical Evidence

### 4.1. Anastomotic Patency Over Time

Long-term patency data following LVA remain surprisingly sparse, particularly for lower extremity lymphedema. Table 1 and Table 2 summarize the available evidence, stratified by extremity.

A striking asymmetry emerges from this comparison. Upper extremity LVA has been evaluated by multiple independent groups, whereas lower extremity data rely predominantly on a single study providing direct long-term assessment. Critically, no study has evaluated lower extremity LVA patency beyond 24 months using standardized methods.

The progressive decline observed in lower extremity patency—from 75% at one year to 36% at two years—aligns with the hemodynamic predictions outlined in Section 2. The time course is more consistent with cumulative repetitive injury than with simple wound healing.

### 4.2. Methodological Limitations

This methodological limitation is specific to patency assessment. While clinical outcome measures—limb volume, circumference, quality of life—are assessed in various positions including upright posture, patency evaluation using ICG lymphography is performed exclusively with patients supine. Researchers thus assess anastomotic function only under optimal hemodynamic conditions, completely ignoring the 16 h daily when patients are upright and gradients are unfavorable.

### 4.3. Patency–Outcome Disconnect and Alternative Explanations

Perhaps the most striking paradox in the lower extremity LVA literature is the inconsistent correlation between anastomotic patency and clinical improvement. Maegawa et al. found no significant difference in limb volume reduction between patients with patent anastomoses (600 ± 969 mL) and those without obvious patency (420 ± 874 mL) [28]. This disconnect was reinforced by a recent multicenter randomized controlled trial comparing LVA combined with complex decongestive therapy (CDT) versus CDT alone in 336 patients with lower extremity lymphedema [29]. Despite adequate statistical power, the study found no significant difference in limb circumference reduction between groups (*p* = 0.129), though LVA did reduce cellulitis frequency [29]. If anastomoses provide meaningful lymphatic drainage, one would expect measurable volume differences—yet this largest RCT to date failed to demonstrate such benefit.

These findings raise a fundamental question: if anastomoses show no volumetric advantage over conservative therapy alone, what accounts for clinical improvements reported in uncontrolled studies? Several alternative mechanisms warrant consideration. First, intensive compression therapy may be the primary driver: postoperative protocols mandate strict compression garment use, and the structured postoperative follow-up may itself improve compliance. Second, selection bias cannot be ignored, as surgical candidates are typically motivated patients with early-stage disease, intact lymphatic contractility, and identifiable functional vessels—characteristics that independently predict favorable outcomes. Third, behavioral modifications accompanying surgical intervention—including enhanced skin care, weight management, and lymphedema self-monitoring—may contribute to disease control independent of anastomotic function. Finally, natural disease variability confounds interpretation: patients typically seek intervention during symptomatic exacerbations, and subsequent improvement may represent regression to the mean rather than treatment effect. These factors, individually or in combination, may account for clinical improvements observed in observational studies—a pattern difficult to reconcile with restored lymphatic drainage but entirely consistent with the hemodynamic limitations outlined in Section 2.

## 5. Knowledge Gaps and Research Priorities

The preceding analysis reveals fundamental gaps in the evidence base for lower extremity LVA. Most critically, no published study has evaluated anastomotic function during upright posture—the very condition when unfavorable pressure gradients are maximal and drainage is theoretically compromised. Virtually all patency assessments occur in supine patients, representing optimal rather than typical hemodynamic conditions. Additionally, as Table 2 illustrates, direct patency data beyond 24 months are essentially non-existent for lower extremity LVA, leaving long-term anastomotic fate unknown. Although recent randomized controlled trials have compared LVA against conservative therapy for both upper and lower extremity lymphedema [29,30], no sham-controlled trial has been conducted to definitively isolate surgical effects from placebo response and intensive postoperative care.

This review has several limitations. As a narrative review, it lacks the systematic methodology of formal meta-analysis, and the included studies exhibit substantial heterogeneity in patient selection, surgical technique, assessment methods, and follow-up duration. Moreover, the relative paucity of lower extremity-specific data necessitated extrapolation from upper extremity studies in some instances.

Future research should prioritize several objectives. First, development of protocols for ambulatory pressure monitoring at recipient venule sites would enable direct assessment of hemodynamic conditions during upright daily activities. Second, standardized upright ICG lymphography could evaluate anastomotic function under physiologically relevant conditions. Third, randomized sham-controlled trials comparing LVA plus compression against sham surgery plus compression are essential to distinguish surgical from non-surgical treatment effects. Fourth, consensus on standardized functional outcome measures—assessed during upright posture rather than exclusively supine—would facilitate meaningful comparison across studies.

## 6. Conclusions

The hemodynamic analysis presented in this review raises a fundamental question: does lower extremity LVA truly provide sustained therapeutic benefit? Adults spend 16–18 h daily in non-supine postures—not only standing but also sitting—during which venous pressure in the lower extremity consistently exceeds lymphatic pressure regardless of whether lymphatic valves are competent or incompetent. This basic physiological reality has never been adequately addressed, yet the surgical community continues to offer LVA to patients with lower extremity lymphedema based largely on supine-position assessments and uncontrolled clinical observations.

This raises not only a scientific concern but an ethical one. If the proposed mechanism of action cannot be justified by elementary hemodynamic principles, is it appropriate to continue performing these procedures without more rigorous evidence? The author respectfully urges proponents of lower extremity LVA to provide direct, posture-specific evidence demonstrating that these anastomoses function during upright and seated daily activities—through ambulatory pressure monitoring, upright ICG lymphography, or randomized sham-controlled trials comparing LVA with conservative therapy alone.

The author acknowledges that this critique is also self-directed. As a surgeon who performs LVA for lower extremity lymphedema, the author confronts this uncertainty with every case—questioning whether the procedure offers genuine physiological benefit or whether patients improve primarily through the intensified conservative management that accompanies surgical intervention. The hope that “doing something is better than doing nothing” may be understandable, but it is not a substitute for evidence. Until such evidence is forthcoming, honest acknowledgment of what remains unknown may serve patients better than confident assertions of efficacy.

## Figures and Tables

**Table 1:** Reported Patency Rates of Upper Extremity LVA.

Study	Year	Anastomosis Type	Follow-Up	Patency Rate	Method
Suzuki et al. [25]	2019	Side-to-end	6 mo	32%	ICG
		End-to-end	6 mo	35%	
Winters et al. [26]	2019	Mixed	12 mo	67% *	ICG
Wolfs et al. [27]	2020	Mixed	12 mo	76% *	ICG

* At least one patent anastomosis per patient. Abbreviation: ICG, indocyanine green lymphography; mo, months.

**Table 2:** Reported Patency Rates of Lower Extremity LVA.

Study	Year	Anastomosis Type	Follow-Up	Patency Rate	Method
Maegawa et al. [28]	2012	Side-to-end	12 mo	75%	ICG
			24 mo	36%	
Multiple studies ^†^ [27]	—	Mixed	6–12 mo	44–75%	Various
>24 mo	—	—	—	No data	—

^†^ Patency range cited in Wolfs et al., 2020 [27], referencing multiple sources.

## Data Availability

No new data were created or analyzed in this study.

## Abbreviations

The following abbreviations are used in this manuscript:

LVA	Lymphovenous anastomosis
ICG	Indocyanine green lymphography
